# 25-Hydroxycholesterol exerts both a cox-2-dependent transient proliferative effect and cox-2-independent cytotoxic effect on bovine endothelial cells in a time- and cell-type-dependent manner

**DOI:** 10.1186/2040-2384-2-24

**Published:** 2010-11-11

**Authors:** Vicky PKH Nguyen, Stephen H Chen, Katerina Pizzuto, Alyssa Cantarutti, Alyssa Terminesi, Cassandra Mendonca, Daniel J Dumont

**Affiliations:** 1 Molecular and Cellular Biology Research, Sunnybrook Research Institute, Sunnybrook Health Sciences Centre, Toronto, ON, M4N 3M5, Canada; 2 Department of Medical Biophysics, University of Toronto, ON, M5G 2M9, Canada; 3 Saint Elizabeth Catholic High School, Toronto, ON, L4J 7X3, Canada

## Abstract

**Background:**

25-hydroxycholesterol (25-OHC) is a product of oxidation of dietary cholesterol present in human plasma. 25-OHC and other oxidized forms of cholesterol are implicated in modulating inflammatory responses involved in development of atherosclerosis and colon carcinogenesis.

**Methods:**

Primary lymphatic, venous and arterial endothelial cells isolated from bovine mesentery (bmLEC, bmVEC, bmAEC) were treated with 25-OHC and tested for several different cellular parameters.

**Results:**

We found 25-OHC to be a potent inducer of cyclooxygenase-2 (Cox-2, prostaglandin G-H synthase-2) expression in bovine mesenteric lymphatic, venous, and arterial endothelial cells. The induction of Cox-2 expression in endothelial cells by 25-OHC led to an initial increase in cellular proliferation that was inhibited by the Cox-2 selective inhibitor celecoxib (Celebrex). Prolonged exposure to 25-OHC was cytotoxic. Furthermore, endothelial cells induced to express Cox-2 by 25-OHC were more sensitive to the effects of the Cox-2 selective inhibitor celecoxib (Celebrex). These results suggest that some effects of 25-OHC on cells may be dependent on Cox-2 enzymatic activity.

**Conclusions:**

Cox-2 dependent elevating effects of 25-OHC on endothelial cell proliferation was transient. Prolonged exposure to 25-OHC caused cell death and enhanced celecoxib-induced cell death in a cell-type dependent manner. The lack of uniform response by the three endothelial cell types examined suggests that our model system of primary cultures of bmLECs, bmVECs, and bmAECs may aid the evaluation of celecoxib in inhibiting proliferation of different types of tumour-associated endothelial cells.

## Background

The enzyme cholesterol-25-hydroxylase (CH25H) converts dietary cholesterol to 25-hydroxycholesterol (25-OHC, cholest-5-ene-3β, 25-diol) in a variety of tissues including heart, lungs, kidney [1,2], and intestinal epithelium [3]. As reviewed by Javitt, 25-OHC only plays a minor role (approximately 5%) in bile acid synthesis in the liver and may play a more active role as a ligand in the regulation of cholesterol synthesis and transport [4]. Indeed, 25-OHC has been detected in blood plasma [5] suggesting that it may have system-wide effects in the body, although the biochemical function of 25-OHC has not been fully elucidated.

Some observations of the effects of 25-OHC include: inhibition of 3-hydroxy-3-methyhydroxy-CoA (HMG-CoA) reductase activity correlating with reduction in mouse cultured fetal liver cell growth [6]; and inhibition of sterol regulatory element-binding proteins (SREBPs) [7]. HMG-CoA reductase and SREBPs are key players in the synthesis of cholesterol and other isoprenoids in the cell--HMG-CoA catalyses the rate-determining step and SREBPs are transcription factors promoting the expression of genes involved in the process [8]. Thus, 25-OHC is thought to attenuate cholesterol and steroid lipid biosynthesis, down-regulation of which is potentially linked to observations that 25-OHC exposure causes cell-cycle arrest and inhibits growth in immortalized and transformed A31 mouse embryonic cells [9], and human primary prostate stromal cells in culture [10].

Potentially unrelated to the role of 25-OHC in regulating cholesterol and isoprenoid synthesis are observations that 25-OHC induces apoptosis in the human acute lymphoblastic leukemia cell line, CEM, by suppression of c-myc expression [11,12], in mouse macrophage-like P388-D1 cells by suppression of the cysteine protease, CPP32 [13], and in hamster ovarian CHO-K1 cells by caspase activation [14]. Likewise, the induction of cyclooxygenase-2 (Cox-2, prostaglandin G-H synthase-2) expression in cultured bovine coronary artery endothelial cells (ECs) does not depend on the activity of Cytochrome P450 (CYP), which are enzymes essential for cholesterol and isoprenoid biosynthesis [15]. Similar observations were noted in rabbit pulmonary arterial ECs and smooth muscle cells (SMCs) exposed to 25-OHC. Treatment with 25-OHC resulted in increased synthesis of eicosanoid products of the arachidonic acid oxidation pathway partly catalyzed by Cox-1 and -2 enzymes [16]. These observations contribute to the idea that 25-OHC play many roles in cell biology that are only beginning to be elucidated.

Whereas 25-OHC treatment leads to induction of Cox-2 expression [15,16], treatment of cells with selective inhibitors to Cox-2 has been shown to induce cell death in endothelial progenitor cells (EPCs) [17], and to induce cell-cycle arrest in ECs [18]. The latter observations would suggest that 25-OHC treatment should promote Cox-2 expression and thereby should contribute to cellular proliferation. However, studies such as by Larsson and colleagues [9] and Wang and colleagues suggest the opposite outcome [10].

In order to understand effects of 25-OHC on cells, we exposed cultured primary bovine lymphatic and blood ECs (bmECs), that do not normally express Cox-2, to 25-OHC. We found 25-OHC to induce Cox-2 expression in primary cultured bovine mesenteric lymphatic, venous, and arterial ECs (bmLECs, bmVECs, bmAECs), correlating with an initial increase in cell count. Exposure of 25-OHC treated bmECs to the Cox-2-selective inhibitor celecoxib (Celebrex) inhibited the observed short burst of increase in cell count. We found bmECs to be able to tolerate short exposures to low levels of 25-OHC. However, prolonged exposure to 25-OHC resulted in cell death, which was more pronounced in bmECs treated with celecoxib. These results suggest some effects of 25-OHC may be dependent upon Cox-2 enzyme activity in ECs.

## Methods

### Cell Culture and 25-OHC Treatment

Primary bmLECs, bmVECs, and bmAECs were from frozen stocks of bmECs previously isolated and described by Nguyen and colleagues [19]. Human colorectal carcinoma cell line HCT-116 was obtained from ATCC (American Type Culture Collection) and ECCC and grown according to provided instructions. BmECs were grown in standard tissue culture conditions in Dulbecco's Modified Eagle's Medium (DMEM, Sigma Aldrich) supplemented with 10% fetal bovine serum (FBS, Sigma Aldrich). For all experiments involving treatment with 25-OHC and/or celecoxib, dialyzed FBS (Gibco). 5 μM Celecoxib was used unless otherwise stated.

In all relevant experiments, the concentration of 25-OHC (dissolved in ethanol (EtOH), Sigma Aldrich) was kept constant at 25 μM as previously described by Wohlfiel and Campbell [15] to be effective for induction of Cox-2 expression. Wherever indicated, short exposure of cells to 25-OHC means at most 24 hours, and prolonged exposure means at least 48 hours.

### Detection of transcripts by RT-PCR

Total RNA was prepared from bmECs using Tri-Reagent (Sigma Aldrich) according to manufacturer's instructions. cDNA was synthesized from 1 μg of total RNA with Thermoscript Reverse Transcriptase (Invitrogen) according to manufacturer's instructions. PCR was performed with Taq polymerase (Qiagen) with primer sequences: *Cox1 *(forward primer 5'-CTGTTGTTACTATCCATGCC-3', reverse primer 5'- CTGGAAAAGCTGCTCATCGC-3'), *Cox2 *(forward primer 5'-GAGAAAACTGCTCAACACCG-3', reverse primer 5'-GCATACTCTGTTGTGTTCCC-3'), *GAPDH *(forward primer 5'-ACC ACA GTC CAT GCC ATC AC-3', reverse primer 5'-TCC ACC ACC CTG TTG CTG TA-3').

### Viable Cell Counting

Cell counts were done using a hemocytometer. Cells were stained with 0.08% Trypan Blue (Gibco) after trypsin (Gibco) treatment. Eight μL of the cell suspension was loaded into each side of the haemocytometer. Four squares with similar cell distribution were counted. Blue (non-viable) cells were excluded from tally. All experiments were done in at least duplicates and repeated at least twice.

### Quantification of Cell Death by ELISA

Cells were incubated with 25 μM 25-OHC and/or celecoxib at various concentrations for at least 60 hours before harvesting. Cell death was quantified using the Cell Death Dectection ELISA-Plus kit (Roche) according to manufacturer's instructions. Amount of coloured immobilized antibodies-histone complexes was determined by spectrophotometric absorbance reading at 405 nm with wavelength correction set at 509 nm.

### Statistical Analysis

Hypothesis testing by appropriate statistical tests was done on all sets of data. P-values were calculated using paired or unpaired, 1 or 2-tailed, student t-test or ANOVA when appropriate, with the confidence interval set at 95% (α = 0.05).

## Results

### 25-OHC induces Cox-2 expression

**Figure 1:**
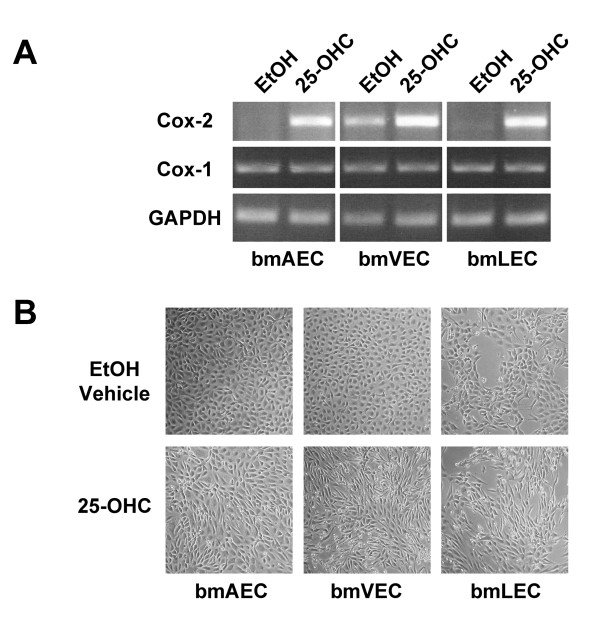
**Expression of Cox2 in mesenteric ECs**. **A/ **25-OHC exposure induced Cox-2 expression in bmECs. ECs were treated with 25 μM of 25-OHC added directly to culture media and left overnight before mRNA harvest and RT-PCR. BmLECs and bmAECs expressed Cox-2 in the presence of 25-OHC but not in EtOH vehicle alone. Cox-1, however, was constitutively expressed in all three cell types. Basal level of Cox-2 was high in bmVECs but not in bmAECs or bmLECs. Cox-2 levels increased significantly upon treatment of bmVECs with 25-OHC. **B/ **25-OHC exposure changed morphology cultured bmECs. ECs lost the typical endothelial cobble-stoned morphology and became elongated, forming partial swirls in the presence of 25-OHC but not in vehicle control.

As previously described by Wohlfeil and Campbell [15,16], 25-OHC stimulates expression of Cox-2 in cultured ECs. Wohlfeil and Campbell exposed bovine coronary arterial cells to a concentration of 10 μg/mL (25 μM) of 25-OHC for 48 hours. To determine whether 25-OHC similarly induces Cox-2 expression our bmLECs, bmVECs, and bmAECs, we treated ECs with 25 μM added directly to culture media and left overnight. By RT-PCR, bmLECs and bmAECs expressed Cox-2 in the presence of 25-OHC but not in ethanol (EtOH) vehicle alone. Cox-1, however, was constitutively expressed in all three cell types (Figure 1A). Interestingly, basal level of Cox-2 was high in bmVECs but not in bmAECs or bmLECs. Cox-2 levels increased significantly upon treatment of bmVECs with 25-OHC. Thus, these results corroborate those reported by Wohlfeil and Campbell [15,16].

Further corroborating results reported by Wohlfeil and Campbell [15], who found 25-OHC to cause cultured rabbit pulmonary arterial ECs and SMCs to take on an elongated morphology, we also found changes in morphology of our cultured bmECs. BmECs lost the typical endothelial cobble-stoned morphology and became elongated, forming partial swirls in the presence of 25-OHC (Figure 1B).

### Short 25-OHC exposure promotes proliferation

**Figure 2:**
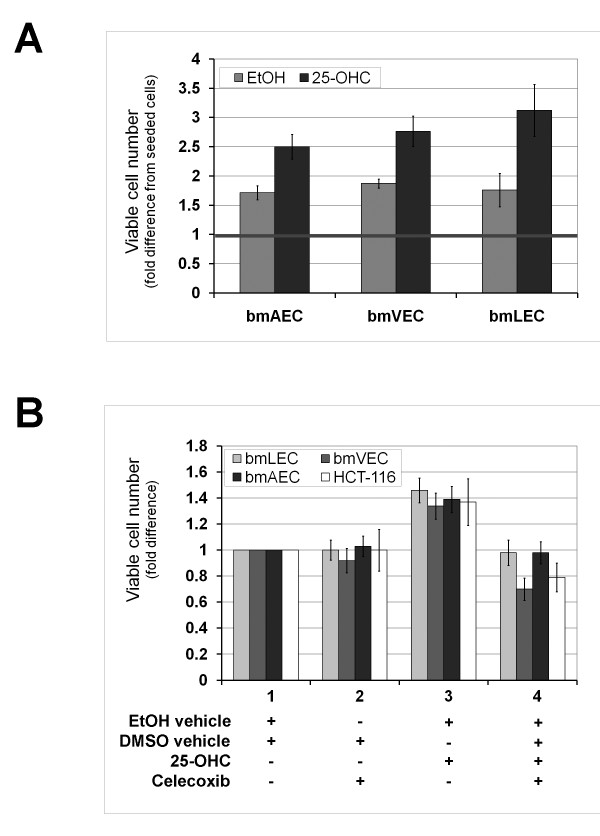
**25-OHC promotes endothelial cell proliferation**. **A/ **Short exposure to 25-OHC promoted EC proliferation. Viable cells that excluded trypan blue were counted after 24 hours of growing cells in 25-OHC. Fold difference in the number of viable cells from seeded cells (taken as 1) was most pronounced in bmLECs compared to bmVECs and bmAECs. Mean of fold differences between treated and untreated bmAEC, bmVEC, and bmLEC from three trials analysed by t-test yielded p-values of 0.021, 0.016, and 0.0036 respectively (n = 3). **B/ **Temporary boost in EC proliferation due to 25-OHC exposure for 12 hours was dependent on Cox-2 activity. ECs were treated with the selective Cox-2 inhibitor celecoxib for 12 hours after 25-OHC exposure. Celecoxib (5 μM) reversed the small increase in cell number of viable cells due to 25-OHC. The number of viable bmVECs treated with celecoxib alone dropped below the number of viable vehicle-treated bmVECs. Proliferative effects of 25-OHC also applied to human colorectal carcinoma cells HCT-116. HCT-116 has previously shown to be Cox-2 deficient. For each cell type, mean of fold differences between treatments from three trials analysed by ANOVA generated p-values ≤ 0.001 (n = 3). For each treatment with celecoxib, 25-OHC, and both together, the mean fold differences between each cell type analysed by ANOVA generated p-values of 0.084, 0.011, and 0.00038, respectively (n = 3).

In addition to the changed morphology, we observed that plates of cells treated with 25-OHC for 24 hours or less appeared to be more tightly packed with cells than those treated with ethanol vehicle alone. We also observed some ECs loosely attached to and growing outside the monolayer (Figure 1b). Without 25-OHC treatment, all three bmECs formed a monolayer in culture with flat cells touching at all sides without squeezing tightly together. This observation led us to hypothesize that perhaps there was an increase in the number of cells packed in the monolayer. Indeed, we found this to be the case when we counted the cells after 24 hours of growing cells in 25-OHC (Figure 2a). Interestingly, the fold difference in the number of viable cells from seeded cells (taken as 1 in figure 2a) seemed most pronounced in bmLECs compared to bmVECs and bmAECs (Figure 2a).

To determine whether the increase in cell count was dependent on Cox-2 enzyme activity, we treated cells with the selective Cox-2 inhibitor celecoxib (Celebrex) after 25-OHC exposure. When cells were treated with 25-OHC in the presence of celecoxib for 12 hours, cell counts were the same as vehicle alone (Figure 2b). Particularly noteworthy was the count of bmVECs treated with celecoxib: the number of viable bmVECs dropped below the count of vehicle treated bmVECs (Figure 2b). bmVECs seemed to be more sensitive to celecoxib in culture media than bmAECs or bmLECs. This observation is in agreement with PCR results shown in figure 1a, where a high basal level of Cox-2 transcript was clearly present in vehicle-treated bmVECs but not in bmAECs or bmLECs; Cox-2 enzyme activity may play a role in the growth of bmVECs in culture.

To determine whether proliferative effects of 25-OHC on cells was restricted ECs, we subjected the human colorectal carcinoma cells HCT-116 to the same treatments as the bmECs (Figure 2b). HCT-116 has previously been shown to be Cox-2 deficient by Molina and colleagues [20]. We found treatment of HCT-116 with 25-OHC increased viable cell count and the increase to be reversed by celecoxib as observed in bmECs (Figure 2b). Therefore the proliferative effects of 25-OHC on cells was not restricted to primary ECs but also applicable to colorectal carcinoma cells in culture.

### Prolonged exposure to 25-OHC treatment caused cell death

**Figure 3:**
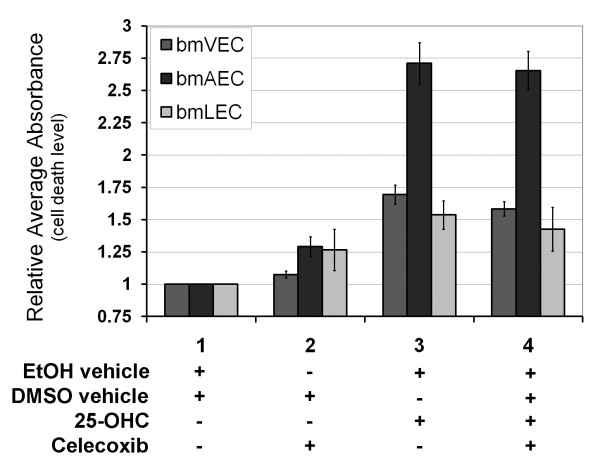
**Long-term exposure to 25-OHC is toxic to ECs**. Exposure to 25-OHC past 60 hours caused the cells to undergo cell death. Levels of EC death due to 25-OHC was measured at 72 hours by ELISA assay quantifying DNA fragmentation. BmAECs were significantly more susceptible to cell death induced by prolonged 25-OHC exposure than either bmVECs or bmLECs. Celecoxib at 5 μM did not reverse the cell death.

Although 25-OHC gave bmECs a boost in cellular proliferation, primary bmECs did not lose contact inhibition. When ECs reached confluency after seeding, they stopped growing even in the presence of 25-OHC. Whether cells were confluent or still proliferating, we observed that continued exposure to 25-OHC past 60 hours caused the cells to undergo cell death (Figure 3). The level of cell death was measured at 72 hours by an ELISA assay quantifying DNA fragmentation. BmAECs were significantly more susceptible to cell death induced by prolonged 25-OHC exposure than either bmVECs or bmLECs. Furthermore, we were not able to rescue the cells by inhibiting Cox-2 activity with 5 μM celecoxib (Figure 3). This result suggests that cell death induced by prolonged 25-OHC exposure was not dependent on Cox-2 enzyme activity.

### 48 hour exposure to 25-OHC sensitizes cells with undetectable basal levels of Cox-2 to effects of Celecoxib

**Figure 4:**
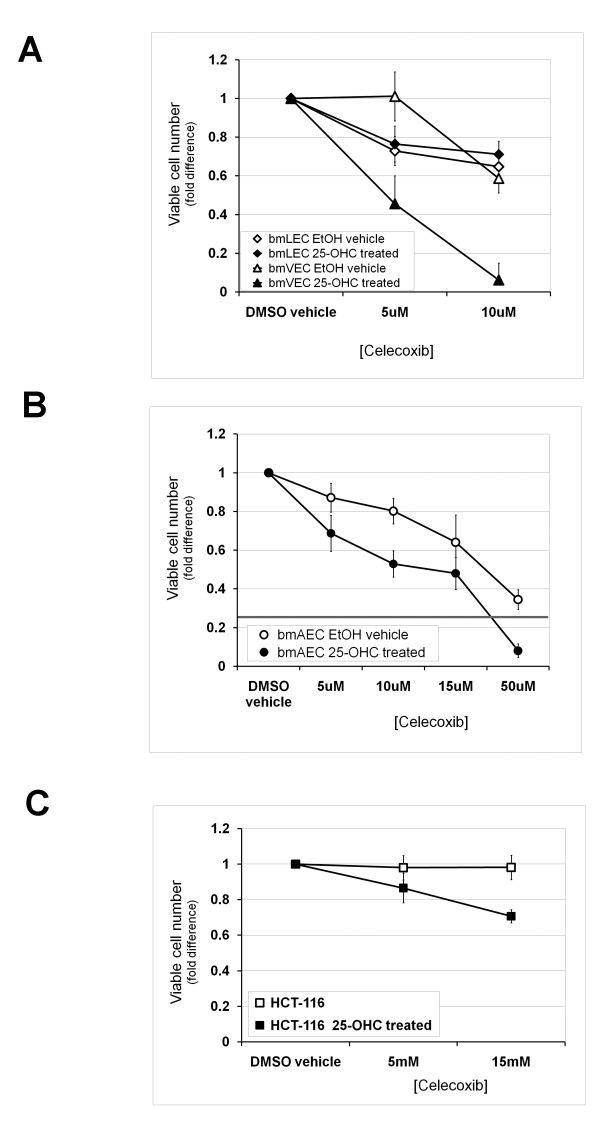
**48 hour exposure to 25-OHC sensitized Cox-2-negative cells to effects of Celecoxib**. **A/ **Celecoxib concentrations of 5 and 10 μM caused the same reduction in viable cell numbers of bmLECs whether or not 25-OHC was present. Unlike bmLECs, viable numbers of bmVECs not treated with 25-OHC did not change as a result of treatment with celecoxib until the concentration was above 5 μM. Mean of fold differences between EtOH-treated (vehicle, 25-OHC untreated) bmLECs and bmVECs in the presence of 5 μM celecoxib analysed by t-test generated p-value of 0.0005 (n = 4). **B/ **Unlike either bmVECs or bmLECs, even 5 μM of celecoxib statistically significantly reduced viable bmAEC cell counts slightly (p = 0.012, n = 4). Cell count was reduced to a greater extent in the presence of 25-OHC. Mean fold differences between 25-OHC treated and 25-OHC untreated bmAECs at 10 μM analysed by t-test generated p-value of 0.0004 (n = 4). **C/ **Low doses of celecoxib also increased reduction in viable cell counts of colorectal carcinoma cells HCT-116 (previously shown to be Cox-2 deficient) in the presence of 25-OHC. Unlike ECs, viable HCT-116 cell numbers did not differ from vehicle control in the absence of 25-OHC. Mean fold difference between cell counts of HCT-116 treated and not treated with 25-OHC analysed by t-test generated p-value of 0.029 at 5 μM celecoxib, and a p-value of 0.0006 at 15 μM (n = 3).

Low doses of celecoxib (5-10 μM) have been shown to induce G2/M cell cycle arrest through inhibition of Cox-2 dependent prostaglandin E2 production [21]. In order to compare the effects of celecoxib on cells with undetectable basal levels of Cox-2 and cells induced to express detectable levels of Cox-2, we performed cell counts with trypan blue after growing cells for a period of 48 hours in the presence of low doses of celecoxib. We chose 48 hours because exposure of bmECs to 25-OHC over this period of time was not long enough to result in toxic effects leading to massive cell death as measured in Figure 3. Furthermore the initial growth advantage conferred on cells by 25-OHC induction of Cox-2 shown in Figure 1 was no longer detectable after 48 hours due to contact inhibition. Low dose celecoxib caused the same reduction in viable bmLEC cell numbers whether or not 25-OHC was present (Figure 4a). Unlike bmLECs, viable numbers of bmVECs not treated with 25-OHC did not change as a result of treatment with celecoxib until the concentration was above 5 μM (Figure 4a). Unlike either bmVECs or bmLECs, even 5 μM of celecoxib reduced viable bmAEC cell counts, which was reduced to a greater extent in the presence of 25-OHC (Figure 4b). Treatment of bmAECs with 50 μM of celecoxib in the presence of 25-OHC caused massive cell death reducing viable cell numbers to below one-fifth of vehicle control. These results indicate that each bmEC type exhibited different levels of sensitivity to celecoxib with or without 25-OHC treatment.

To determine whether low doses of celecoxib also increased reduction in viable cell counts of colorectal carcinoma cells with undetectable basal Cox-2 expression, we performed the same experiment on colorectal carcinoma cells HCT-116 previously shown to be Cox-2 deficient [20]. Unlike ECs, in the absence of 25-OHC, viable HCT-116 cell numbers did not differ from vehicle control even at 10 μM of celecoxib (Figure 4c). In the presence of 25-OHC, HCT-116 viable cell numbers reduced as celecoxib concentrations increased from 5 μM to 15 μM (Figure 4c). These results indicate that increased sensitivity to effects of celebrex as a result of 25-OHC exposure was not restricted to primary ECs with undetectable basal levels of Cox-2 but also applicable to Cox-2-deficient colorectal carcinoma cells in culture.

## Discussion

We set out in this study to elucidate the effects of 25-OHC on mammalian cells and to determine whether those effects are dependent on Cox-2. We found as previously reported by Wohlfeil and Campbell [15,16] that 25-OHC induces Cox-2 expression. We found that short exposure to 25-OHC to induce Cox-2 led to a temporary increase in viable cell numbers that was reversed by the Cox-2 inhibitor celecoxib. Low dose of celecoxib used (5 μM) ensured that non-specific binding of celecoxib to other targets was minimized--celecoxib is selective for Cox-2 at 5 μM, below previously reported IC_50 _value of 15 μM for celecoxib binding to Cox-1 (reviewed in [22]). Contrastingly, the addition of celecoxib did not prevent cell death following prolonged exposure to 25-OHC. These results suggest that not all effects of 25-OHC on cells were dependent on Cox-2 activity.

Bovine cells have previously been established as acceptable model cells for studies involving Cox-2 inhibitors intended for human use. For example, Jung et al (2007) used bovine aortic endothelial cells to demonstrate anti-cancer properties of the Cox-2 inhibitor enoic acanthoic acid [23]. For another example, Toker et al (2008) demonstrated the relaxant effects of celecoxib on bovine ciliary muscle [24]. Most significantly, Myers et al (2010) demonstrated that bovine cells respond to non-steroidal anti-inflammatory drugs (NSAIDs) intended for human use similarly to other mammalian cells in suppressing Cox-2 activity [25]. Bovine ECs are therefore highly suitable cell models for studies such as described herein.

25-OHC is detected in human plasma at concentrations ranges of 0 to 11 ng/mL [5], however, cultured testicular macrophages have been shown to be capable of producing high levels of 25-OHC comparable to the quantity chosen in the present study (10 μg/mL) [26]. Nes et al (2000) reported cultured testicular macrophages were capable of producing 10 fg 25-OHC per cell per hour [26]. Therefore media concentration of 25-OHC can reach 10 μg/mL in approximately 4 days assuming 50 million cells grown in 5 mL of media. Furthermore, 10 μg/mL was shown by other members of the same laboratory to be ineffective in causing cytotoxicity of Leydig cells, in which 25-OHC is metabolized to form testosterone [27]. Only when Lukyanenko et al (2001) used a concentration of 100 μg/mL over an exposure period of 2 days did they observe toxicity to 50% of Leydig cells [27]. To a lesser extent, the bovine ECs in the present study were observed to be resistant to 25-OHC cytotoxic effects at 10 μg/mL over an exposure period of 1 day.

Previously, 25-OHC treatment was shown to increase cell death, increase prostacyclin production (suggestive of cyclooxygenase activity induction), and decrease proliferation when compared to cholesterol treatment [28]. The cytotoxicity of 25-OHC on cultured human umbilical arterial ECs was demonstrated by Kawamura and Kummerow to be both concentration and time dependent [28]. We have performed a similar examination of 25-OHC cytotoxicity on arterial as well as venous and lymphatic ECs and found that not all EC types were equally affected by 25-OHC exposure and/or celecoxib treatment. BmAECs had the highest level of cell death upon 25-OHC exposure (Figure 3). The effect of celecoxib on bmLECs did not seem to be dependent on 25-OHC exposure (Figure 4a). Furthermore, cells with undetectable levels of endogenous basal Cox-2 like bmAECs and HCT-116 were further sensitized to celecoxib if they were previously exposed to 25-OHC (Figure 4b and 4c). Our results seem to suggest that effects of 25-OHC may be cell-type dependent in addition to being partially dependent on the status of Cox-2 expression by the cell.

Our results in Figure 1a corroborate work by others' work establishing 25-OHC as an inducer of Cox-2 expression in ECs [15,16]. The fact that during inflammation, Cox-2 expression in ECs is induced by cytokines such as interleukin-1β (IL-1β) [29] released by macrophages, suggests that 25-OHC may be pro-inflammatory. However, evidence exists indicating that 25-OHC may be anti-inflammatory. This evidence comes from studies of the Liver X Receptor (LXR), for which 25-OHC is an agonistic ligand [30]. For example, synthetic LXR agonists have been shown to inhibit IL-1β-induced production of Prostaglandin E2 (PGE_2_), as well as Cox-2 in osteoarthritic cartilage and in the synovial sarcoma cell line SW982 [31]. For another example, LXR agonists such as GW3965 inhibited expression of pro-inflammatory mediators such as nitric oxide (NO) synthase, Cox-2, and IL-6 in macrophages [32]. Although LXRs are also expressed by ECs [33], Morello et al (2009) established that pro-inflammatory effects of 25-OHC in ECs are not dependent on LXRs [33]. Altogether these studies indicate that 25-OHC may act as an anti-inflammatory agent in an LXR-dependent manner in certain tissues and a pro-inflammatory agent in an LXR-independent manner in other tissues, most notably ECs.

Indirectly, 25-OHC has been shown to induce immune cells such as macrophages to express and secrete interleukin-1β (IL-1β) [34], which induces Cox-2 expression in ECs [29] as well as in colon cancer cells [35]. Macrophages and other immune cells are frequently recruited to the site of tumourigenesis by factors secreted by colon cancer cells [36]. Macrophages at the site of tumourigenesis have been shown to activate colon cancer cells by increasing their proliferation and metastasis potential. Activated colon cancer cells in turn stimulate blood ECs to undergo angiogenesis [37]. Moreover, 25-OHC in combination with IL-1β has been shown to stimulate human colon carcinoma cells (Caco-2) to produce IL-8 [38], which in turn is a promoter of angiogenesis by stimulating endothelial cells proliferation, survival, migration, and MMP-2 production [39,40]. Taken together, these results may suggest that 25-OHC at concentrations that are not cytotoxic may participate in colon tumourigenesis with or without Cox-2 involvement, although 25-OHC may cooperate with some tumourigenic effects of Cox-2.

Since 25-OHC is an LXR activating ligand, and since LXR has recently been suggested to inhibit proliferation of breast cancer [41], it logically follows that 25-OHC should perhaps be considered to be anti-tumourigenic. However we suggested in the previous paragraph that 25-OHC may be pro-tumourigenic when it is not cytotoxic. Indeed, we observed that short-term exposure to 25-OHC stimulated EC proliferation, which could be reversed by celecoxib (Figure 2). The apparent conflict can be resolved when the contexts in which effects of 25-OHC on cells are considered. Results by Morello et al (2009) using human umbilical venous endothelial cells led the investigators to suggest that, at least in EC, 25-OHC was not as potent as other oxysterols such as 22-OHC or 24,25-OHC in activating LXR [33]. The suggestion by these same investigators that perhaps more prolonged exposure of cells to 25-OHC would lead to more dramatic LXR-dependent effects was partly corroborated by our results indicating more prolonged exposure of ECs to 25-OHC was required to cause cytotoxicity (Figure 3). All together, these findings suggest that not all effects of 25-OHC on cells are LXR-dependent and mechanistically linked to the induction of Cox-2 and thereby PGE_2 _production. Thus, 25-OHC may still be considered a potential cytotoxic agent with anti-cancer properties despite certain pro-inflammatory characteristics such as the induction of Cox-2 expression.

In health, Cox-2 is normally not constitutively detectable in vascular endothelial cells and epithelial cells of the gastrointestinal tract and is more commonly detected in parts of the brain, kidney, pancreatic islet, ovary and in uterine cells (reviewed in [42]). Cox-2 expression is induced in the event of injury by tissues requiring damage repair and at sites of inflammation (reviewed in [42]). In colon cancer, Cox-2 has been found to be abnormally over-expressed in about 90% of colorectal adenocarcinomas and in 40-90% of colorectal adenomas (reviewed in [43]). The abnormal over-expression of Cox-2 is not only found in tumour cells but also in almost every cell type in the surrounding tumour including fibroblasts, myofibroblasts, mononuclear inflammatory cells, and most importantly, endothelial cells (reviewed in [43]). Expression of Cox-2 in ECs surrounding colorectal tumours has been shown to promote angiogenesis and lymphangiogenesis by mechanisms dependent on PGE_2 _production. Products of the enzymatic pathway perform such tasks as promotion of EC proliferation, survival, increasing EC motility towards the tumour, and up-regulation of VEGF-C levels [44]. Expression of Cox-2 in ECs can also promote tumourigenesis by mechanisms independent of PGE_2 _production such as activation of carcinogens [45] and reduction in arachidonic levels [46]. These tumourigenic properties of Cox-2 are part of the reason why cyclooxygenase inhibitors are under review as a potential cancer therapy particularly for colon cancer [47].

Previously, Penning et al (1997) established 15 μM to be the half maximal inhibitory concentration (IC50) of celecoxib for Cox-1 and 0.04 μM. In this study, we chose to examine effects of low celecoxib concentrations (5 - 15 μM) in order to minimize off-target effects of celecoxib and ensure that most of our observations were due to Cox-2 inhibition. While the choice of dose did allow us to observe some Cox-2-dependent effects, we also found the viability of Cox-2-deficient HCT-116 to be reduced even at these low concentrations (Figure 4c). These results are expected considering that other investigators have demonstrated anti-proliferative effects of celecoxib to extend beyond Cox-2 inhibition in colon cancer cell lines [48], hematopoietic and epithelial cell lines [49] and prostate cancer xenografts [50].

## Conclusions

Cox-2-dependent effects of 25-OHC on endothelial cell proliferation was transient. Prolonged exposure to 25-OHC caused cell death and enhanced celecoxib-induced cell death in a less Cox-2 dependent manner. More importantly, the three EC types represented by the bovine mesenteric ECs did not respond identically to the Cox-2 inhibitor celecoxib--bmLECs and bmAECs but not bmVECs were sensitive to even 5 uM of celecoxib (Figures 3 and 4). Thus, Cox-2 inhibitors as anti-cancer treatments may show cell-type selectivity. Three-cell models such as our primary cultures of bmLECs, bmVECs, and bmAECs can aid examination of the effectiveness of celecoxib on proliferation/cell cycle of different tumour associated ECs. In future experiments, our three EC types extracted from bovine mesentery may also be treated with immune cytokines such as IL-1β and IL-8, in addition to tumourigenic compounds such as 25-OHC to recapitulate activated ECs in the colon tumour environment.

## Competing interests

The authors declare that they have no competing interests.

All authors have read and approved the final manuscript.

## Authors' contributions

VPKHN conceived the ideas, performed experiments, analysed data, produced figures and wrote the paper; KP contributed to writing the paper and contributed work to experiments; SC performed experiments requiring PCR; AC, AT, and CM contributed work to experiments; DJD contributed to the writing and the development of the project.
